# Chlorido[2,15-dimethyl-3,7,10,14,20-penta­azabicyclo­[14.3.1]eicosa-1(20),2,14,16,18-penta­ene]manganese(II) perchlorate acetonitrile solvate

**DOI:** 10.1107/S1600536809005595

**Published:** 2009-02-21

**Authors:** Agnieszka Głowińska, Violetta Patroniak, Wanda Radecka-Paryzek, Maciej Kubicki

**Affiliations:** a Department of Chemistry, Adam Mickiewicz University, Grunwaldzka 6, 60-780 Poznań, Poland

## Abstract

The Mn ion in the title complex, [MnCl(C_17_H_27_N_5_)]ClO_4_·CH_3_CN, is six-coordinated with a geometry inter­mediate between penta­gonal pyramidal and heavily distorted octa­hedral. In the macrocycle, the pyridinium ring makes a large dihedral angle of 63.70 (9)° with the best plane through the remaining four N atoms. This feature is common for 17-membered N_5_ rings, in contrast to their 16- and 15-membered analogues which often form planar N_5_ systems. In the crystal, N—H⋯O and C—H⋯O interactions help to establish the packing. The perchlorate counter-ion is rotationally disordered around the chlorine centre, with occupation factors of 0.74 (1) and 0.26 (1).

## Related literature

For manganese(II) metalloproteins and penta­aza macrocyclic complexes, see, for example: Riley (1999 ▶); Aston *et al.* (2001 ▶); Patroniak *et al.* (2004 ▶); Radecka-Paryzek *et al.* (2005 ▶); Isobe *et al.* (2005 ▶); Grabolle *et al.* (2006 ▶). For the crystal structures of similar 17-membered macrocycles, see: Drew *et al.* (1977 ▶, 1979 ▶); Nelson *et al.* (1977 ▶); Drew & Nelson (1979 ▶).
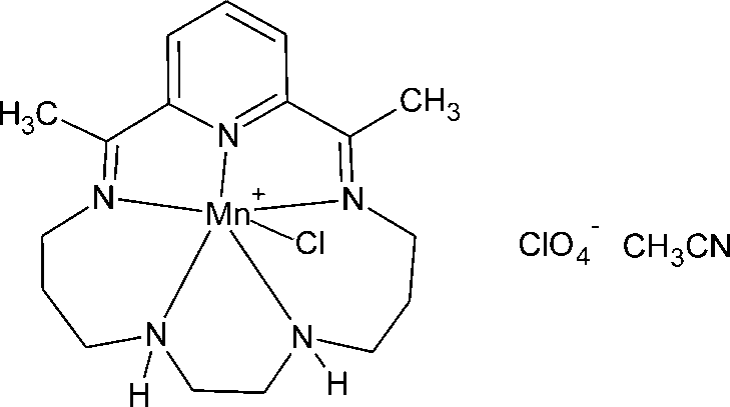


## Experimental

### 

#### Crystal data

[MnCl(C_17_H_27_N_5_)]ClO_4_·C_2_H_3_N
*M* *_r_* = 532.33
Triclinic, 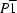

*a* = 10.0583 (7) Å
*b* = 10.9118 (7) Å
*c* = 11.9591 (8) Å
α = 89.492 (5)°
β = 70.195 (6)°
γ = 84.093 (5)°
*V* = 1227.89 (14) Å^3^
*Z* = 2
Mo *K*α radiation
μ = 0.79 mm^−1^
*T* = 293 K
0.4 × 0.2 × 0.1 mm

#### Data collection

Kuma KM-4 CCD diffractometer
Absorption correction: multi-scan (*CrysAlis CCD*; Oxford Diffraction, 2007 ▶) *T*_min_ = 0.842, *T*_max_ = 0.924
9805 measured reflections
4301 independent reflections
3212 reflections with *I* > 2σ(*I*)
*R*_int_ = 0.018

#### Refinement

*R*[*F* ^2^ > 2σ(*F* ^2^)] = 0.042
*wR*(*F* ^2^) = 0.132
*S* = 1.12
4301 reflections
329 parameters
68 restraints
H-atom parameters constrained
Δρ_max_ = 0.43 e Å^−3^
Δρ_min_ = −0.43 e Å^−3^

### 

Data collection: *CrysAlis CCD* (Oxford Diffraction, 2007 ▶); cell refinement: *CrysAlis RED* (Oxford Diffraction, 2007 ▶); data reduction: *CrysAlis RED*; program(s) used to solve structure: *SHELXS97* (Sheldrick, 2008 ▶); program(s) used to refine structure: *SHELXL97* (Sheldrick, 2008 ▶); molecular graphics: *Stereochemical Workstation Operation Manual* (Siemens, 1989 ▶); software used to prepare material for publication: *SHELXL97*.

## Supplementary Material

Crystal structure: contains datablocks I, global. DOI: 10.1107/S1600536809005595/bg2236sup1.cif

Structure factors: contains datablocks I. DOI: 10.1107/S1600536809005595/bg2236Isup2.hkl

Additional supplementary materials:  crystallographic information; 3D view; checkCIF report

## Figures and Tables

**Table 1:** Selected bond lengths (Å)

Mn1—N20	2.234 (2)
Mn1—N3	2.326 (3)
Mn1—N7	2.327 (3)
Mn1—N10	2.336 (3)
Mn1—N14	2.350 (3)
Mn1—Cl1	2.3934 (9)

**Table 2:** Hydrogen-bond geometry (Å, °)

*D*—H⋯*A*	*D*—H	H⋯*A*	*D*⋯*A*	*D*—H⋯*A*
C25—H252⋯O2^i^	0.96	2.27	3.168 (6)	156
N7—H7⋯O1	0.91	2.16	3.050 (6)	165
N10—H10⋯O3*A*^i^	0.91	2.23	3.13 (2)	169

Symmetry code: (i) 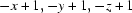.

**Fig. 1.**
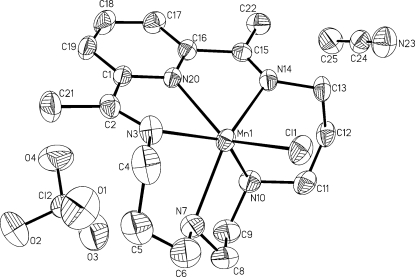
Anisotropic displacement ellipsoid representation (at the 50% probability level) of the asymmetric unit content. Only the larger fraction of the disordered perchlorate is shown.

## supplementary crystallographic information

### Comment

The significance of metal complexes containing synthetic macrocyclic ligands is most obvious as it relates to naturally occurring macrocyclic systems such as the porphyrin core in hemoglobin or chlorophylls, the corrin in vitamin B_12_, cyclic polyether antibiotics. Many of the recent advances in the coordination chemistry of manganese have arisen from the desire to understand and mimic the mechanism of water oxidation and dioxygen evolution during photosynthesis catalyzed by manganese metalloproteins (Grabolle *et al.*, 2006; Isobe *et al.*, 2005). The manganese(II) pentaaza macrocyclic complexes have been considered as synzymes (low molecular weight catalysts which mimic a natural enzymatic function) for superoxide anion dismutation and activity with the goal to design and synthesis better human pharmaceutical agents (Riley, 1999; Aston *et al.*, 2001). The effective method for the synthesis of macrocyclic complexes involves the coordination template effect. It consists of a metal ion being used to orient the reacting groups of linear substrates in the desired conformation for the condensation process which ultimately ends with ring closure (Radecka-Paryzek *et al.*, 2005). We have recently reported the first examples of 16-membered macrocyclic lanthanide complexes which are able to activate molecular oxygen (Patroniak *et al.*, 2004). Here we present the template action of manganese(II) in the synthesis of 17-membered pentaaza macrocycle. The crystal structure of the perchlorate salt of the resulting complex, chloro-(2,15-dimethyl-3,7,10,14,20-pentaazabicyclo-[14.3.1]eicosa-1(20),2,14,16,18-pentaene)-manganese(ii), 1, which crystallizes as the acetonitrile solvate, reveals that this metal ion which has no crystal-field stabilization energy in the high-spin state, can be accommodated by the particular stereochemical constraints enforced by the template process and adopt rare coordination geometry.

The N_5_-system in the 17-membered quinquedentate macrocyclic ligand does not form a plane, as it is often a case for 15- and 16-membered analogues (*e.g.* Patroniak *et al.*, 2004). Four non-pyridine nitrogen atoms N3, N7, N10 and N14 are approximately coplanar - however even for these four atoms the maximum deviation from the least-squares plane is as high as 0.207 (2) Å - and the pyridine nitrogen N20 is 1.369 (3)Å out of this plane (Fig. 1). The pyridine ring makes a dihedral angle of 63.70 (9)° with the mean plane of the remaining N_4_-system; a similar value was found in the thiocyanato-lead complex (63.1°, Drew & Nelson, 1979), while it was smaller, but still significant, in other complexes: 48.8° for bromo-mercury (Drew *et al.*, 1979), 49.7° for bromo-cadmium (Drew *et al.*, 1979), and 41.8° for bis-isothiocyanato-manganese (Drew *et al.*, 1977).

This non-planar disposition of five nitrogen atoms results also in an uncommon coordination of the Mn ion, which can be described as intermediate between a heavily distorted pentagonal pyramid (with the Cl atom at the apex and the N_5_ system as the base) and a distorted octahedron (*cf.* Table 1).

The perchlorate counterions are disordered over two positions with site occcupation factors of 0.74 (1) and 0.26 (1).

Through very weak interactions between cation and anions (See Table 2 for the most relevant ones), a centrosymmetric tetramer is formed around the cell centre, which appears as the building block on which the crystal architecture is based. Two solvent - acetonitrile molecules join to these tetramers by means of a rather linear C—H···O hydrogen bond. These cation-anion groups further organize into columns along the [001] direction, probably through second order contacts involving the Chlorine atoms (H···Cl ~2.90Å) which might impose some directionality to the main driving force of the crystal packing, the coulombic interaction between charged fragments.

### Experimental

To a mixture of MnCl_2_.4H_2_O (0.065 g, 0.32 mmol) and Mn(ClO_4_)_2_. 6H_2_O (0.059 g, 0.16 mmol) in methanol (5 ml), 4,7-diazadecane-1,10-diamine (0.090 ml, 0.48 mmol) in methanol (5 ml) and 1,2-diacetylpyridine (0.081 g, 0.048 mmol) in methanol (5 ml) was added dropwise with stirring. The reaction was carried out for 24 h under reflux at argon atmosphere. The reaction mixture was evaporated to dryness and the remaining solid dissolved in boiling acetonitrile (15 ml), filtered under gravity, and left to stand overnight. Crystals suitable for X-ray diffraction analysis were formed.

ESI-MS m/*z* (%) = 171 (100 {[Mn*L*^2^]}^2+^);377 (33 {[Mn*L*^2^](Cl)}^+^); 441 (39 {[Mn*L*^2^](ClO_4_)}^+^).

Elemental analysis calculated for [Mn*L*^2^Cl](ClO_4_).6H_2_O: C, 32.83; H, 6.37; N,11.96; found: C, 33.29; H, 4.72; N, 6.7.

### Refinement

Hydrogen atoms were located geometrically and refined in the 'riding model', with *U*_iso_'s set at 1.2 (1.5 for methyl groups) times *U*_eq_'s of their appropriate carrier atoms. Weak restraints were applied to both the geometry (*DFIX* for Cl—O bond lengths and O···O 1,3-distances) and displacement parameters (ISOR) of O atoms from the disordered perchlorate group.

### Crystal data

**Table:**

[MnCl(C_17_H_27_N_5_)]ClO_4_·C_2_H_3_N	*Z* = 2
*M**_r_* = 532.33	*F*(000) = 554
Triclinic, *P*1	*D*_x_ = 1.440 Mg m^−^^3^
Hall symbol: -P 1	Mo *K*α radiation, λ = 0.71073 Å
*a* = 10.0583 (7) Å	Cell parameters from 5931 reflections
*b* = 10.9118 (7) Å	θ = 3–24°
*c* = 11.9591 (8) Å	µ = 0.79 mm^−^^1^
α = 89.492 (5)°	*T* = 293 K
β = 70.195 (6)°	Block, colourless
γ = 84.093 (5)°	0.4 × 0.2 × 0.1 mm
*V* = 1227.89 (14) Å^3^	

### Data collection

**Table:**

Kuma KM-4 CCD diffractometer	4301 independent reflections
Radiation source: fine-focus sealed tube	3212 reflections with *I* > 2σ(*I*)
graphite	*R*_int_ = 0.018
ω scans	θ_max_ = 25.0°, θ_min_ = 2.7°
Absorption correction: multi-scan (*CrysAlis CCD*; Oxford Diffraction, 2007)	*h* = −11→8
*T*_min_ = 0.842, *T*_max_ = 0.924	*k* = −12→12
9805 measured reflections	*l* = −14→12

### Refinement

**Table:**

Refinement on *F*^2^	Primary atom site location: structure-invariant direct methods
Least-squares matrix: full	Secondary atom site location: difference Fourier map
*R*[*F*^2^ > 2σ(*F*^2^)] = 0.042	Hydrogen site location: inferred from neighbouring sites
*wR*(*F*^2^) = 0.132	H-atom parameters constrained
*S* = 1.12	*w* = 1/[σ^2^(*F*_o_^2^) + (0.08*P*)^2^] where *P* = (*F*_o_^2^ + 2*F*_c_^2^)/3
4301 reflections	(Δ/σ)_max_ < 0.001
329 parameters	Δρ_max_ = 0.43 e Å^−^^3^
68 restraints	Δρ_min_ = −0.43 e Å^−^^3^

### Special details

**Table:**

Geometry. All e.s.d.'s (except the e.s.d. in the dihedral angle between two l.s. planes) are estimated using the full covariance matrix. The cell e.s.d.'s are taken into account individually in the estimation of e.s.d.'s in distances, angles and torsion angles; correlations between e.s.d.'s in cell parameters are only used when they are defined by crystal symmetry. An approximate (isotropic) treatment of cell e.s.d.'s is used for estimating e.s.d.'s involving l.s. planes.
Refinement. Refinement of *F*^2^ against ALL reflections. The weighted *R*-factor *wR* and goodness of fit *S* are based on *F*^2^, conventional *R*-factors *R* are based on *F*, with *F* set to zero for negative *F*^2^. The threshold expression of *F*^2^ > σ(*F*^2^) is used only for calculating *R*-factors(gt) *etc*. and is not relevant to the choice of reflections for refinement. *R*-factors based on *F*^2^ are statistically about twice as large as those based on *F*, and *R*- factors based on ALL data will be even larger.

### Fractional atomic coordinates and isotropic or equivalent isotropic displacement parameters (Å^2^)

**Table:**

	*x*	*y*	*z*	*U*_iso_*/*U*_eq_	Occ. (<1)
Mn1	0.44426 (5)	0.28963 (4)	0.20642 (4)	0.04702 (19)	
Cl1	0.31564 (10)	0.17792 (9)	0.11320 (8)	0.0692 (3)	
C1	0.7488 (3)	0.3700 (3)	0.1391 (2)	0.0476 (7)	
C2	0.7747 (3)	0.2331 (3)	0.1285 (3)	0.0544 (8)	
C21	0.9208 (4)	0.1739 (4)	0.1132 (4)	0.0838 (12)	
H21A	0.9209	0.0859	0.1134	0.109*	
H21B	0.9492	0.2002	0.1774	0.109*	
H21C	0.9862	0.1976	0.0390	0.109*	
N3	0.6681 (3)	0.1794 (2)	0.1317 (2)	0.0579 (7)	
C4	0.6780 (5)	0.0432 (4)	0.1191 (4)	0.0882 (13)	
H4A	0.7750	0.0114	0.0738	0.106*	
H4B	0.6173	0.0215	0.0759	0.106*	
C5	0.6331 (5)	−0.0152 (3)	0.2403 (5)	0.0938 (14)	
H5A	0.6526	−0.1040	0.2289	0.113*	
H5B	0.6910	0.0110	0.2842	0.113*	
C6	0.4797 (5)	0.0153 (4)	0.3139 (5)	0.0972 (15)	
H6A	0.4210	−0.0044	0.2682	0.117*	
H6B	0.4571	−0.0350	0.3838	0.117*	
N7	0.4460 (3)	0.1468 (3)	0.3517 (3)	0.0682 (8)	
H7	0.5139	0.1655	0.3813	0.082*	
C8	0.3099 (4)	0.1695 (4)	0.4485 (4)	0.0905 (13)	
H8A	0.3115	0.1198	0.5158	0.109*	
H8B	0.2339	0.1468	0.4227	0.109*	
C9	0.2845 (5)	0.3013 (4)	0.4839 (3)	0.0799 (11)	
H9A	0.3599	0.3232	0.5109	0.096*	
H9B	0.1953	0.3167	0.5492	0.096*	
N10	0.2796 (3)	0.3788 (3)	0.3822 (2)	0.0589 (7)	
H10	0.3047	0.4543	0.3933	0.071*	
C11	0.1369 (3)	0.3957 (4)	0.3697 (3)	0.0662 (9)	
H11A	0.0650	0.4035	0.4482	0.079*	
H11B	0.1234	0.3230	0.3304	0.079*	
C12	0.1163 (3)	0.5084 (4)	0.2992 (3)	0.0635 (9)	
H12A	0.1351	0.5800	0.3367	0.076*	
H12B	0.0175	0.5204	0.3045	0.076*	
C13	0.2087 (3)	0.5036 (4)	0.1681 (3)	0.0592 (9)	
H13A	0.1856	0.4369	0.1272	0.071*	
H13B	0.1904	0.5802	0.1315	0.071*	
N14	0.3594 (2)	0.4841 (2)	0.1571 (2)	0.0478 (6)	
C15	0.4326 (3)	0.5735 (3)	0.1512 (2)	0.0469 (7)	
C22	0.3868 (4)	0.7083 (3)	0.1474 (3)	0.0668 (10)	
H22A	0.2947	0.7186	0.1391	0.087*	
H22B	0.4540	0.7437	0.0810	0.087*	
H22C	0.3825	0.7487	0.2197	0.087*	
C16	0.5821 (3)	0.5369 (3)	0.1479 (3)	0.0476 (7)	
C17	0.6801 (4)	0.6185 (3)	0.1400 (3)	0.0631 (9)	
H17A	0.6559	0.7028	0.1380	0.076*	
C18	0.8150 (4)	0.5727 (4)	0.1352 (3)	0.0704 (10)	
H18A	0.8816	0.6263	0.1340	0.084*	
C19	0.8505 (3)	0.4478 (4)	0.1321 (3)	0.0654 (10)	
H19A	0.9423	0.4160	0.1254	0.078*	
N20	0.6156 (2)	0.4155 (2)	0.1509 (2)	0.0425 (5)	
N23	−0.0126 (5)	0.8132 (4)	0.1994 (4)	0.1062 (13)	
C24	0.0164 (4)	0.8829 (4)	0.2528 (4)	0.0760 (11)	
C25	0.0549 (6)	0.9727 (4)	0.3213 (5)	0.1053 (16)	
H251	0.1320	1.0136	0.2699	0.137*	
H252	0.0837	0.9318	0.3821	0.137*	
H253	−0.0254	1.0322	0.3574	0.137*	
Cl2	0.72849 (11)	0.28375 (9)	0.48084 (8)	0.0727 (3)	
O1	0.7114 (7)	0.2002 (6)	0.4036 (5)	0.155 (3)	0.744 (7)
O2	0.8302 (5)	0.2290 (5)	0.5320 (5)	0.136 (2)	0.744 (7)
O3	0.6031 (5)	0.3181 (7)	0.5761 (4)	0.156 (3)	0.744 (7)
O4	0.7887 (6)	0.3879 (5)	0.4240 (5)	0.149 (3)	0.744 (7)
O1A	0.6571 (17)	0.3333 (16)	0.4029 (12)	0.150 (8)	0.256 (7)
O2A	0.706 (2)	0.1585 (7)	0.491 (2)	0.194 (10)	0.256 (7)
O3A	0.668 (2)	0.3472 (18)	0.5903 (10)	0.202 (13)	0.256 (7)
O4A	0.8736 (8)	0.297 (2)	0.429 (2)	0.45 (4)	0.256 (7)

### Atomic displacement parameters (Å^2^)

**Table:**

	*U*^11^	*U*^22^	*U*^33^	*U*^12^	*U*^13^	*U*^23^
Mn1	0.0438 (3)	0.0503 (3)	0.0490 (3)	−0.0094 (2)	−0.0170 (2)	−0.0083 (2)
Cl1	0.0697 (6)	0.0756 (6)	0.0717 (6)	−0.0229 (5)	−0.0314 (5)	−0.0163 (5)
C1	0.0384 (15)	0.068 (2)	0.0364 (15)	−0.0069 (14)	−0.0123 (12)	−0.0001 (14)
C2	0.0488 (18)	0.070 (2)	0.0444 (17)	0.0035 (16)	−0.0181 (14)	−0.0054 (15)
C21	0.053 (2)	0.102 (3)	0.093 (3)	0.014 (2)	−0.027 (2)	0.005 (2)
N3	0.0594 (17)	0.0550 (16)	0.0611 (17)	0.0056 (13)	−0.0254 (14)	−0.0202 (13)
C4	0.096 (3)	0.065 (2)	0.113 (4)	0.017 (2)	−0.053 (3)	−0.039 (2)
C5	0.119 (4)	0.040 (2)	0.138 (4)	−0.003 (2)	−0.065 (3)	−0.007 (2)
C6	0.107 (4)	0.053 (2)	0.145 (4)	−0.022 (2)	−0.057 (3)	0.013 (3)
N7	0.070 (2)	0.0633 (18)	0.078 (2)	−0.0174 (15)	−0.0311 (17)	0.0106 (16)
C8	0.080 (3)	0.098 (3)	0.089 (3)	−0.020 (3)	−0.021 (2)	0.037 (3)
C9	0.084 (3)	0.098 (3)	0.048 (2)	−0.009 (2)	−0.0101 (19)	0.002 (2)
N10	0.0586 (16)	0.0675 (18)	0.0475 (15)	−0.0065 (14)	−0.0139 (13)	−0.0032 (13)
C11	0.0507 (19)	0.084 (3)	0.053 (2)	−0.0094 (18)	−0.0022 (16)	−0.0102 (18)
C12	0.0380 (17)	0.090 (3)	0.056 (2)	0.0052 (17)	−0.0099 (15)	−0.0180 (18)
C13	0.0404 (16)	0.081 (2)	0.058 (2)	0.0041 (16)	−0.0216 (15)	−0.0095 (17)
N14	0.0386 (13)	0.0638 (17)	0.0399 (13)	0.0000 (12)	−0.0133 (11)	−0.0075 (12)
C15	0.0493 (17)	0.0554 (18)	0.0340 (15)	−0.0048 (14)	−0.0118 (13)	−0.0024 (13)
C22	0.068 (2)	0.056 (2)	0.066 (2)	0.0073 (17)	−0.0138 (18)	0.0016 (17)
C16	0.0473 (17)	0.0570 (19)	0.0406 (15)	−0.0078 (14)	−0.0169 (13)	−0.0062 (13)
C17	0.067 (2)	0.064 (2)	0.062 (2)	−0.0224 (18)	−0.0226 (18)	−0.0012 (17)
C18	0.060 (2)	0.089 (3)	0.074 (2)	−0.035 (2)	−0.0295 (19)	−0.001 (2)
C19	0.0417 (18)	0.099 (3)	0.060 (2)	−0.0172 (19)	−0.0211 (16)	0.005 (2)
N20	0.0366 (12)	0.0502 (14)	0.0414 (13)	−0.0061 (11)	−0.0134 (10)	−0.0028 (11)
N23	0.105 (3)	0.085 (3)	0.138 (4)	−0.012 (2)	−0.052 (3)	−0.015 (3)
C24	0.065 (2)	0.062 (2)	0.102 (3)	−0.0037 (19)	−0.031 (2)	0.009 (2)
C25	0.130 (4)	0.081 (3)	0.125 (4)	−0.022 (3)	−0.066 (4)	0.007 (3)
Cl2	0.0845 (7)	0.0778 (6)	0.0613 (6)	−0.0233 (5)	−0.0277 (5)	−0.0020 (5)
O1	0.182 (5)	0.162 (5)	0.141 (5)	−0.044 (4)	−0.071 (4)	−0.066 (4)
O2	0.143 (4)	0.144 (4)	0.146 (5)	0.012 (3)	−0.088 (4)	0.017 (3)
O3	0.107 (4)	0.252 (7)	0.081 (3)	0.034 (4)	−0.012 (3)	−0.019 (4)
O4	0.164 (5)	0.132 (4)	0.169 (5)	−0.070 (4)	−0.064 (4)	0.062 (4)
O1A	0.168 (11)	0.172 (12)	0.119 (9)	−0.018 (8)	−0.060 (8)	0.029 (8)
O2A	0.227 (14)	0.160 (13)	0.204 (14)	−0.056 (9)	−0.074 (10)	0.006 (9)
O3A	0.226 (16)	0.211 (15)	0.181 (15)	−0.026 (10)	−0.081 (10)	−0.055 (9)
O4A	0.44 (4)	0.45 (4)	0.46 (4)	−0.054 (12)	−0.142 (16)	0.024 (11)

### Geometric parameters (Å, °)

**Table:**

Mn1—N20	2.234 (2)	C11—H11A	0.9700
Mn1—N3	2.326 (3)	C11—H11B	0.9700
Mn1—N7	2.327 (3)	C12—C13	1.526 (4)
Mn1—N10	2.336 (3)	C12—H12A	0.9700
Mn1—N14	2.350 (3)	C12—H12B	0.9700
Mn1—Cl1	2.3934 (9)	C13—N14	1.469 (3)
C1—N20	1.341 (4)	C13—H13A	0.9700
C1—C19	1.376 (4)	C13—H13B	0.9700
C1—C2	1.489 (5)	N14—C15	1.269 (4)
C2—N3	1.263 (4)	C15—C22	1.500 (4)
C2—C21	1.496 (4)	C15—C16	1.502 (4)
C21—H21A	0.9600	C22—H22A	0.9600
C21—H21B	0.9600	C22—H22B	0.9600
C21—H21C	0.9600	C22—H22C	0.9600
N3—C4	1.485 (5)	C16—N20	1.337 (4)
C4—C5	1.520 (6)	C16—C17	1.374 (4)
C4—H4A	0.9700	C17—C18	1.379 (5)
C4—H4B	0.9700	C17—H17A	0.9300
C5—C6	1.498 (6)	C18—C19	1.370 (5)
C5—H5A	0.9700	C18—H18A	0.9300
C5—H5B	0.9700	C19—H19A	0.9300
C6—N7	1.478 (5)	N23—C24	1.118 (5)
C6—H6A	0.9700	C24—C25	1.446 (7)
C6—H6B	0.9700	C25—H251	0.9600
N7—C8	1.463 (5)	C25—H252	0.9600
N7—H7	0.9100	C25—H253	0.9600
C8—C9	1.475 (6)	Cl2—O1	1.367 (3)
C8—H8A	0.9700	Cl2—O2	1.438 (3)
C8—H8B	0.9700	Cl2—O3	1.403 (4)
C9—N10	1.487 (4)	Cl2—O4	1.403 (3)
C9—H9A	0.9700	Cl2—O1A	1.429 (5)
C9—H9B	0.9700	Cl2—O2A	1.406 (5)
N10—C11	1.485 (4)	Cl2—O3A	1.398 (5)
N10—H10	0.9100	Cl2—O4A	1.402 (5)
C11—C12	1.521 (5)		
			
N20—Mn1—N3	68.62 (9)	C9—N10—Mn1	109.3 (2)
N20—Mn1—N7	118.71 (9)	C11—N10—H10	108.4
N3—Mn1—N7	75.97 (10)	C9—N10—H10	108.4
N20—Mn1—N10	105.07 (9)	Mn1—N10—H10	108.4
N3—Mn1—N10	141.85 (9)	N10—C11—C12	113.0 (3)
N7—Mn1—N10	75.11 (10)	N10—C11—H11A	109.0
N20—Mn1—N14	68.58 (8)	C12—C11—H11A	109.0
N3—Mn1—N14	130.50 (9)	N10—C11—H11B	109.0
N7—Mn1—N14	148.65 (10)	C12—C11—H11B	109.0
N10—Mn1—N14	73.60 (9)	H11A—C11—H11B	107.8
N20—Mn1—Cl1	137.26 (7)	C11—C12—C13	115.8 (3)
N3—Mn1—Cl1	100.45 (7)	C11—C12—H12A	108.3
N7—Mn1—Cl1	96.45 (8)	C13—C12—H12A	108.3
N10—Mn1—Cl1	107.03 (7)	C11—C12—H12B	108.3
N14—Mn1—Cl1	94.43 (7)	C13—C12—H12B	108.3
N20—C1—C19	120.6 (3)	H12A—C12—H12B	107.4
N20—C1—C2	114.3 (3)	N14—C13—C12	109.6 (2)
C19—C1—C2	125.1 (3)	N14—C13—H13A	109.7
N3—C2—C1	114.9 (3)	C12—C13—H13A	109.7
N3—C2—C21	127.0 (3)	N14—C13—H13B	109.7
C1—C2—C21	118.2 (3)	C12—C13—H13B	109.7
C2—C21—H21A	109.5	H13A—C13—H13B	108.2
C2—C21—H21B	109.5	C15—N14—C13	121.8 (3)
H21A—C21—H21B	109.5	C15—N14—Mn1	118.56 (19)
C2—C21—H21C	109.5	C13—N14—Mn1	117.3 (2)
H21A—C21—H21C	109.5	N14—C15—C22	127.6 (3)
H21B—C21—H21C	109.5	N14—C15—C16	114.6 (3)
C2—N3—C4	121.2 (3)	C22—C15—C16	117.7 (3)
C2—N3—Mn1	118.2 (2)	C15—C22—H22A	109.5
C4—N3—Mn1	118.7 (2)	C15—C22—H22B	109.5
N3—C4—C5	110.8 (3)	H22A—C22—H22B	109.5
N3—C4—H4A	109.5	C15—C22—H22C	109.5
C5—C4—H4A	109.5	H22A—C22—H22C	109.5
N3—C4—H4B	109.5	H22B—C22—H22C	109.5
C5—C4—H4B	109.5	N20—C16—C17	121.2 (3)
H4A—C4—H4B	108.1	N20—C16—C15	114.4 (3)
C6—C5—C4	114.7 (4)	C17—C16—C15	124.4 (3)
C6—C5—H5A	108.6	C16—C17—C18	118.7 (3)
C4—C5—H5A	108.6	C16—C17—H17A	120.7
C6—C5—H5B	108.6	C18—C17—H17A	120.7
C4—C5—H5B	108.6	C19—C18—C17	119.7 (3)
H5A—C5—H5B	107.6	C19—C18—H18A	120.2
N7—C6—C5	112.2 (3)	C17—C18—H18A	120.2
N7—C6—H6A	109.2	C18—C19—C1	119.3 (3)
C5—C6—H6A	109.2	C18—C19—H19A	120.3
N7—C6—H6B	109.2	C1—C19—H19A	120.3
C5—C6—H6B	109.2	C16—N20—C1	120.3 (2)
H6A—C6—H6B	107.9	C16—N20—Mn1	120.06 (18)
C8—N7—C6	111.9 (3)	C1—N20—Mn1	118.8 (2)
C8—N7—Mn1	107.3 (2)	N23—C24—C25	179.6 (6)
C6—N7—Mn1	117.4 (3)	C24—C25—H251	109.5
C8—N7—H7	106.6	C24—C25—H252	109.5
C6—N7—H7	106.6	H251—C25—H252	109.5
Mn1—N7—H7	106.6	C24—C25—H253	109.5
N7—C8—C9	109.1 (3)	H251—C25—H253	109.5
N7—C8—H8A	109.9	H252—C25—H253	109.5
C9—C8—H8A	109.9	O3A—Cl2—O4A	112.0 (8)
N7—C8—H8B	109.9	O1—Cl2—O3	113.0 (4)
C9—C8—H8B	109.9	O1—Cl2—O4	112.7 (4)
H8A—C8—H8B	108.3	O3—Cl2—O4	111.0 (4)
C8—C9—N10	110.6 (3)	O3A—Cl2—O2A	111.6 (7)
C8—C9—H9A	109.5	O4A—Cl2—O2A	110.8 (8)
N10—C9—H9A	109.5	O3A—Cl2—O1A	108.0 (7)
C8—C9—H9B	109.5	O4A—Cl2—O1A	108.5 (7)
N10—C9—H9B	109.5	O2A—Cl2—O1A	105.7 (7)
H9A—C9—H9B	108.1	O1—Cl2—O2	108.9 (4)
C11—N10—C9	113.3 (3)	O3—Cl2—O2	106.4 (3)
C11—N10—Mn1	109.01 (19)	O4—Cl2—O2	104.1 (3)
			
N20—C1—C2—N3	0.2 (4)	C9—N10—C11—C12	158.7 (3)
C19—C1—C2—N3	−176.7 (3)	Mn1—N10—C11—C12	−79.3 (3)
N20—C1—C2—C21	179.2 (3)	N10—C11—C12—C13	66.3 (4)
C19—C1—C2—C21	2.2 (5)	C11—C12—C13—N14	−57.5 (4)
C1—C2—N3—C4	178.4 (3)	C12—C13—N14—C15	−92.3 (4)
C21—C2—N3—C4	−0.5 (5)	C12—C13—N14—Mn1	70.2 (3)
C1—C2—N3—Mn1	−17.4 (4)	N20—Mn1—N14—C15	−16.4 (2)
C21—C2—N3—Mn1	163.8 (3)	N3—Mn1—N14—C15	−48.2 (2)
N20—Mn1—N3—C2	19.7 (2)	N7—Mn1—N14—C15	94.0 (3)
N7—Mn1—N3—C2	−109.3 (2)	N10—Mn1—N14—C15	97.6 (2)
N10—Mn1—N3—C2	−67.6 (3)	Cl1—Mn1—N14—C15	−155.9 (2)
N14—Mn1—N3—C2	51.5 (3)	N20—Mn1—N14—C13	−179.5 (2)
Cl1—Mn1—N3—C2	156.6 (2)	N3—Mn1—N14—C13	148.72 (19)
N20—Mn1—N3—C4	−175.6 (3)	N7—Mn1—N14—C13	−69.2 (3)
N7—Mn1—N3—C4	55.3 (3)	N10—Mn1—N14—C13	−65.5 (2)
N10—Mn1—N3—C4	97.0 (3)	Cl1—Mn1—N14—C13	40.94 (19)
N14—Mn1—N3—C4	−143.9 (2)	C13—N14—C15—C22	−3.5 (5)
Cl1—Mn1—N3—C4	−38.8 (3)	Mn1—N14—C15—C22	−165.8 (2)
C2—N3—C4—C5	94.4 (4)	C13—N14—C15—C16	176.4 (2)
Mn1—N3—C4—C5	−69.7 (4)	Mn1—N14—C15—C16	14.0 (3)
N3—C4—C5—C6	65.7 (5)	N14—C15—C16—N20	0.5 (4)
C4—C5—C6—N7	−67.4 (5)	C22—C15—C16—N20	−179.6 (3)
C5—C6—N7—C8	−164.2 (4)	N14—C15—C16—C17	179.1 (3)
C5—C6—N7—Mn1	71.1 (4)	C22—C15—C16—C17	−1.0 (4)
N20—Mn1—N7—C8	122.6 (3)	N20—C16—C17—C18	−0.1 (5)
N3—Mn1—N7—C8	178.2 (3)	C15—C16—C17—C18	−178.6 (3)
N10—Mn1—N7—C8	23.4 (3)	C16—C17—C18—C19	3.4 (5)
N14—Mn1—N7—C8	27.0 (4)	C17—C18—C19—C1	−2.7 (5)
Cl1—Mn1—N7—C8	−82.6 (3)	N20—C1—C19—C18	−1.2 (5)
N20—Mn1—N7—C6	−110.5 (3)	C2—C1—C19—C18	175.5 (3)
N3—Mn1—N7—C6	−54.9 (3)	C17—C16—N20—C1	−3.9 (4)
N10—Mn1—N7—C6	150.3 (3)	C15—C16—N20—C1	174.8 (2)
N14—Mn1—N7—C6	153.9 (3)	C17—C16—N20—Mn1	165.5 (2)
Cl1—Mn1—N7—C6	44.3 (3)	C15—C16—N20—Mn1	−15.8 (3)
C6—N7—C8—C9	178.6 (3)	C19—C1—N20—C16	4.5 (4)
Mn1—N7—C8—C9	−51.3 (4)	C2—C1—N20—C16	−172.5 (3)
N7—C8—C9—N10	60.4 (4)	C19—C1—N20—Mn1	−165.0 (2)
C8—C9—N10—C11	85.5 (4)	C2—C1—N20—Mn1	17.9 (3)
C8—C9—N10—Mn1	−36.3 (4)	N3—Mn1—N20—C16	171.1 (2)
N20—Mn1—N10—C11	125.9 (2)	N7—Mn1—N20—C16	−129.6 (2)
N3—Mn1—N10—C11	−159.6 (2)	N10—Mn1—N20—C16	−48.6 (2)
N7—Mn1—N10—C11	−117.8 (2)	N14—Mn1—N20—C16	16.6 (2)
N14—Mn1—N10—C11	64.2 (2)	Cl1—Mn1—N20—C16	89.0 (2)
Cl1—Mn1—N10—C11	−25.5 (2)	N3—Mn1—N20—C1	−19.29 (19)
N20—Mn1—N10—C9	−109.7 (2)	N7—Mn1—N20—C1	40.0 (2)
N3—Mn1—N10—C9	−35.3 (3)	N10—Mn1—N20—C1	121.0 (2)
N7—Mn1—N10—C9	6.6 (2)	N14—Mn1—N20—C1	−173.8 (2)
N14—Mn1—N10—C9	−171.5 (2)	Cl1—Mn1—N20—C1	−101.4 (2)
Cl1—Mn1—N10—C9	98.9 (2)		

### Hydrogen-bond geometry (Å, °)

**Table:**

*D*—H···*A*	*D*—H	H···*A*	*D*···*A*	*D*—H···*A*
C25—H252···O2^i^	0.96	2.27	3.168 (6)	156
N7—H7···O1	0.91	2.16	3.050 (6)	165
N10—H10···O3A^i^	0.91	2.23	3.13 (2)	169

Symmetry codes: (i) −*x*+1, −*y*+1, −*z*+1.
